# Protein-cell conjugates as artificial surface display for interfacial biocatalysis†

**DOI:** 10.1039/d4sc08063g

**Published:** 2025-02-11

**Authors:** Xiankun Wu, Henrik Karring, Zhongkai Wang, Changzhu Wu

**Affiliations:** a Anhui Provincial Engineering Center for High Performance Biobased Nylons, School of Materials and Chemistry, Anhui Agricultural University Hefei Anhui 230036 China wangzk6@ahau.edu.cn; b Department of Physics, Chemistry and Pharmacy, University of Southern Denmark Campusvej 55 5230 Odense Denmark wu@sdu.dk; c Department of Green Technology, University of Southern Denmark Campusvej 55 5230 Odense Denmark; d Danish Institute for Advanced Study (DIAS), University of Southern Denmark Campusvej 55 5230 Odense Denmark

## Abstract

Interfacial whole-cell biocatalysis has great potential for advanced chemical synthesis due to its ability to efficiently mediate complex reactions. However, the practical use of this approach is often limited by the fragility of living cells and the difficulty of maintaining enzyme activity under interfacial conditions. Here, we propose an artificial surface display strategy for interfacial biocatalysis by directly coupling sodium caseinate (NaCas) to the surface of *E. coli* cells. This coupling creates a robust biointerface that provides two main benefits: protecting cells from harsh interfacial environments and enabling the formation of Pickering emulsions for catalysis. The resulting protein-cell conjugates demonstrated thermal stability and strong resistance to organic solvents. Furthermore, the direct attachment of additional enzymes onto the cell surface allowed for efficient multienzyme cascade reactions, achieving an 80% yield in benzoin synthesis. The platform also showed multienzyme recyclability, retaining over 80% of enzyme activity after five reuse cycles, with emulsions that remained stable for more than 24 hours, enabling long-term catalytic applications. Therefore, these features demonstrate the significant benefits of our artificial surface display strategy, providing an environmentally friendly and versatile platform for interfacial biocatalysis applicable to synthetic chemistry and industrial biotechnology.

## Abstract 2

We developed an artificial interface display strategy that overcomes the longstanding challenges of cellular fragility and enzyme instability in whole-cell interfacial catalysis by engineered living *E. coli* cells with interface-active proteins.

## Graphical abstract

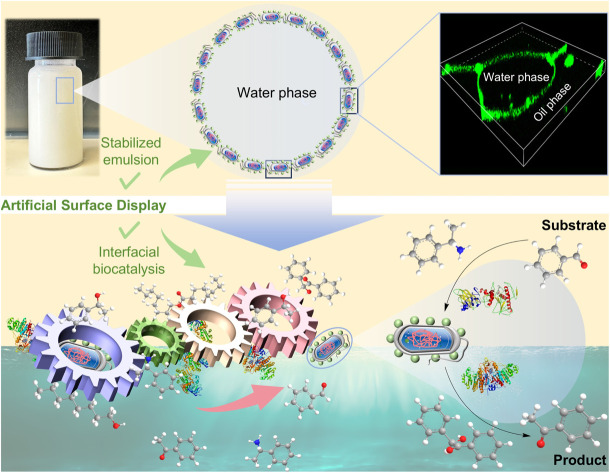

## 1 Introduction

Biocatalysis remains a vital tool in industrial chemistry, offering unparalleled selectivity and sustainability.^1^ More than 60% of industrial biocatalytic processes employ whole-cell systems, primarily due to their ability to bypass the need for complex protein purification.^2–4^ However, whole-cell catalysis is not without its limitations.^5^ The cell membrane often acts as a diffusion barrier, impeding the access of substrates to intracellular enzymes.^6–8^ Additionally, the accumulation of reaction products within the cells can inhibit further catalytic activity, reducing the overall efficiency of the process.^9,10^ These challenges have driven extensive research into cell engineering techniques aimed at optimizing enzyme accessibility and performance in chemical biotransformations. One particularly promising approach is cell surface display, where enzymes are genetically anchored to the outer membrane of cells.^11–13^ This configuration eliminates internal diffusion constraints and enables the construction of multi-enzyme systems on the same surface, facilitating sequential reactions. However, traditional surface display methods face several technical challenges, including protein misfolding and complex preparation procedures, which are associated with high costs.^14^

To address these challenges, artificial surface display for displaying functional molecules in unnatural environments has gained significant attention as a simple and controllable strategy.^15^ Unlike natural systems that rely on biological linkages, artificial surface display involves the covalent or physical attachment of chemical catalysts to the cell membrane. For example, Ward and colleagues have developed methods for attaching small molecule catalysts to cells using supramolecular avidin–biotin interactions.^16^ Similarly, metallic nanoparticles have been employed to functionalize cell surfaces, enhancing catalytic processes.^17^ While these systems have shown improved catalytic performance, they remain limited to chemical catalysts rather than enzymatic ones. Enzymes, when attached to the cell surface, offer a fundamental advantage due to their high catalytic specificity, selectivity, and sustainability.^18,19^ Moreover, enzyme-based systems are inherently more environmentally friendly and cost-effective, aligning with the principles of green chemistry.^20–22^

Interfacial reactions represent another frontier in biocatalysis, particularly because they offer a large surface area for rapid reactions between polar and non-polar substrates at liquid–liquid interfaces.^23–25^ This has led to the development of emulsion biocatalysis, an emerging field where enzymes or enzyme-containing carriers form Pickering interfacial biocatalysts, which exhibit enhanced activity compared to traditional biphasic systems.^26–29^ However, the use of living cells in interfacial catalysis has been historically constrained by their structural fragility and the challenge of maintaining enzyme activity in harsh interfacial environments.^30–32^ Our recent work provided the example of chemically modifying live *E. coli* cells with polydopamine particles to enhance their robustness in these challenging conditions.^33^ While the polydopamine coating effectively protected the cells, it introduced additional preparation steps and mass transfer limitations, thereby reducing the versatility of enzyme display on the cell surface.

**Fig. 1:**
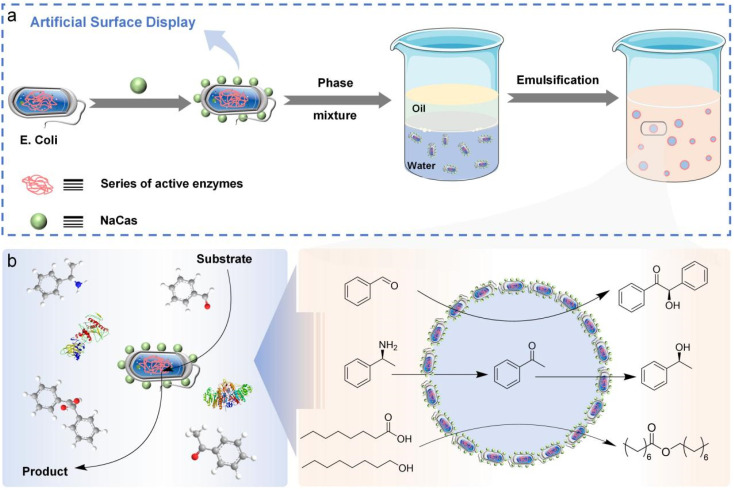
Strategy for the construction of protein-cell-conjugate-based artificial surface display. (a) Building a robust Pickering interfacial biocatalysis (PIB) platform with artificial surface display technology. (b) Interfacial biocatalytic processes stabilized by protein-cell conjugates.

In this proof-of-concept study, we expand on our foundational work by directly attaching functional proteins and enzymes to the surface of *E. coli* cells, creating an artificial surface display that supports robust cellular systems for interfacial biocatalysis. Unlike previous studies, this method avoids cumbersome organic synthesis processes and is non-biotoxic. Sodium caseinate (NaCas) as a green biomass feedstock is directly coupled to cell membranes by amide coupling reaction (Fig. 1a and b). This approach establishes a dynamic biointerface with dual functions: it protects living cells from harsh interfacial conditions while enabling them to form Pickering emulsions at the water-organic interface. A key advantage of this direct protein-cell conjugation strategy is that it eliminates the need for additional protective coatings or external stabilizers, simplifying the system and enhancing its practicality for emulsion catalysis. Moreover, this method allows for the attachment of multiple enzymes to the cell surface, enabling the multienzyme cascades in combination with the intracellular enzymes present in *E. coli* for advanced synthesis. The relatively large size of *E. coli* cells also acts as a “solid scaffold”, contributing to the recyclability of the multienzyme systems and making them a sustainable option for repeated use. Therefore, our conjugate platform offers a versatile, efficient, and environmentally friendly solution for interfacial biocatalysis, paving the way for advancements in synthetic chemistry and industrial biotechnology.

## 2 Results and discussion

### 2.1 Formation of protein-cell conjugates

**Fig. 2:**
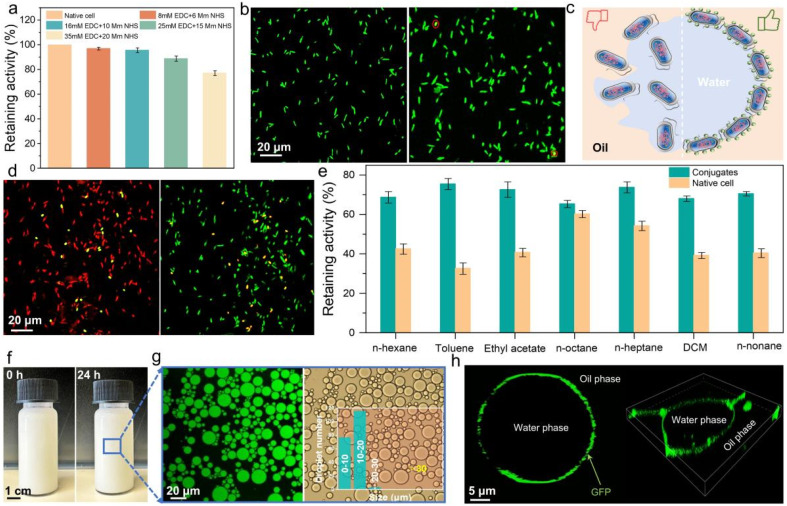
Proof of concept for protein-cell conjugates. (a) Effect of the concentration of coupling agents on the BAL enzymatic retaining activity. (b) Live/dead assay images for native cells and conjugate cells; green-live, red-dead. (c) Scheme of native cells (left) and conjugate cells (right) under interfacial stress (*i.e.*, toluene–water biphasic condition). (d) Live/dead assay of native cells (left) and conjugate cells (right) treated by 1 h interfacial stress; green-live cells and red-dead cells. (e) Protein-cell conjugates exhibit excellent resistance in a wide range of solvents. (f) Emulsion appearance at 0 h and 24 h optical microscopy image of the emulsions. (g) Confocal laser scanning microscopy (CLSM) image of the water-in-oil emulsions; green-aqueous phase (left); optical microscopy image of the emulsions (right); number and distribution of emulsion droplets when the water-to-oil ratio is 5 : 5 (inset). (h) Scheme of emulsion droplet stabilized by conjugate cells with green fluorescent protein (GFP) (left); 3D-CLSM image of emulsion droplet stabilized by conjugate cells containing GFP (right).

First, we purchased *E. coli* BL21 (DE3) cells from Thermo Scientific and cultivated it through preculture and main culture to obtain a large quantity of fresh cells. To design protein-cell conjugates between *E. coli* and NaCas, the coupling reagents, 1-(3-dimethylaminopropyl)-3-ethylcarbodiimide (EDC) and *N*-hydroxy succinimide (NHS), were used for an amide coupling reaction in buffer solution (Fig. S1a†). After the reaction, the conjugates were purified through gentle centrifugation, a mild process aimed to preserve cell functions. To confirm the success of the conjugation, NaCas was first labeled with the green fluorescent dye—fluorescein isothiocyanate (FITC) and then reacted with the cells (Fig. S1b†). Under a confocal laser scanning microscope (CLSM), green fluorescence was observed only on the conjugate surface but not on control samples, indicating a successful and effective conjugation (Fig. S2†). Following this confirmation, we investigated the effect of the coupling agent concentration on the intracellular enzyme activity. For this, we overexpressed the enzyme benzaldehyde lyase (BAL) in *E. coli*. As shown in Fig. 2a and Table S1†, the increase of coupling agents decreased the retaining activity of the enzyme, which may be due to the formation of more amide bonds on the surface of NaCas and the cells. These increased bond formations and agents could potentially induce mass-transfer limitations across cell membranes.^34^ Meanwhile, the biotoxicity of excess chemicals might be another reason for reduced enzyme activity. However, using a low amount of coupling agents was also suboptimal, as it did not provide sufficient NaCas on the cell surface to create an effective interface-active conjugate, which resulted in interfacial destabilization. (Fig. S3†). Based on these results, we consider that 16 mM EDC and 10 mM NHS may be able to achieve synergy between multiple factors to ensure optimal catalytic efficiency. Subsequently, we evaluated the effect of NaCas on cell viability by performing live/dead assays, staining the native cells and conjugated cells with SYTO 9 and propidium iodide, respectively. As shown in Fig. 2b, the conjugated cells retained more than 95% cell viability. Furthermore, quantitative characterization (Fig. S4†) demonstrated that the catalytic activity of the conjugate cells was almost identical to that of native cells, validating the biocompatibility of our selected conjugation strategy.

Encouraged by these promising results, we next explored the protective effects of the NaCas coating in harsh environments (Fig. 2c). To test this, we exposed both native and conjugate cells to 55 °C for 1 hour, followed by live/dead assays. As shown in Fig. 2d, the survival rate of native cells dropped below 10%, while conjugate cells maintained a survival rate above 70%. This significant difference prompted us to explore the impact of temperature on intracellular enzyme activity of cells more closely. We examined BAL activity at three temperatures: 37 °C, 45 °C, and 55 °C. While there was no notable change in activity at 37 °C over time, we observed a marked decline in activity at both 45 °C and 55 °C (Fig. S5†). After 1 hour of incubation at 55 °C, the activity of the protein-cell conjugates was 1.61 times higher than that of native cells, demonstrating enhanced enzyme thermal stability in conjugates.

In addition to thermal stability, we were also interested in the protective effects of the artificial surface display against organic solvents, which are commonly encountered in Pickering interfacial biocatalysis applications. As demonstrated in Fig. 2e, the protein-cell conjugates exhibited strong resistance to organic solvents, consistently showing higher activity than native cells. Meanwhile, the protein-cell conjugate was able to form emulsions with seven organic solvents and remained stable for 12 hours (Fig. S6†). In particular, the enzyme activity of the conjugate cells in toluene was 2.31 times higher than that of the native cells. To get more insight into this case, we evaluated the conjugates' tolerance in toluene over time. Fig. S7† shows that although the activity of both conjugate and native cells decreased over time, the conjugate cells maintained 3 times higher activity than the native cells after 3 hours.

Overall, the robustness and protective effects of the artificial display make protein-cell conjugates a promising platform for further applications in hashing conditions, *e.g.*, Pickering interfacial biocatalysis.

### 2.2 Artificial display-based Pickering emulsions

A typical Pickering emulsion is prepared by mixing two solutions, such as 5 mL of aqueous protein-cell conjugates and 5 mL of toluene. Remarkably, stable Pickering emulsions can be formed simply by hand-shaking the mixture, without the need for external forces like homogenization or vortexing (Fig. 2f). This gentle preparation method prevents damage to the living cells from interface stress.^32^ Even more impressively, the emulsions remained stable for 24 hours without demulsifying, indicating the potential for long-term, stable catalytic applications.

Since the stability of the PIB system depends on the water-to-oil ratio,^35^ we gradually reduced the organic phase from 1 : 9 to 8 : 2 to evaluate its impact on emulsion stability (Fig. S8†). Our findings showed that a 5 : 5 ratio provided the most stable emulsions. To determine the type of emulsion, we used a water-soluble green fluorescent dye, fluorescein isothiocyanate. CLSM images (Fig. 2g and S9†) confirmed the formation of a water-in-oil (W/O) emulsion. At the optimal 5 : 5 ratio, the droplets had the smallest particle size and a uniform distribution (Fig. 2g, inset, and S10†). The particle size distribution was around 20 μm. Furthermore, we calculated the interfacial area of the droplets, which is directly related to the efficiency of interfacial biocatalysis.^7,36–38^ The largest interfacial area was observed at the 5 : 5 water-to-oil ratio (Fig. S11†).

To further investigate the distribution of cells within the emulsion, we prepared emulsions using *E. coli* cells overexpressing green fluorescent protein (GFP). The 2D and 3D CLSM images showed that the fluorescent cells were primarily located on the surface of the droplets, confirming that the emulsions were stabilized by the protein-cell conjugates (Fig. 2h). These results motivate us to further explore the catalytic performance of these artificial surface display in interfacial catalysis.

### 2.3 Interfacial biocatalysis

**Fig. 3:**
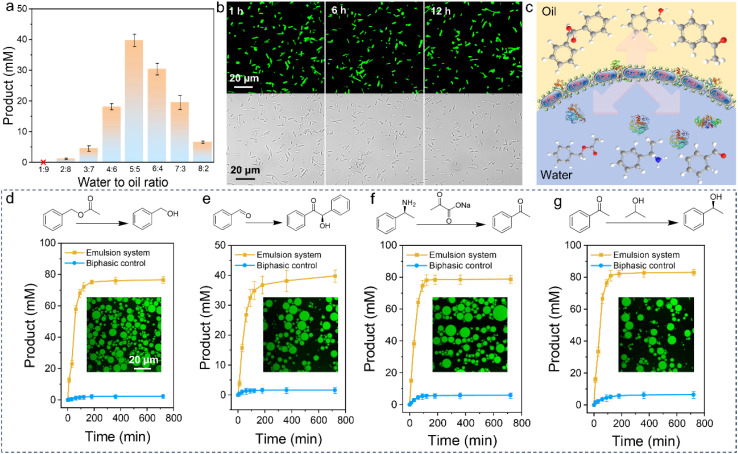
Investigation of single-step reaction in protein-cell conjugates emulsion. (a) BAL enzyme activity of emulsions with different water-to-oil ratios. (b) Examination of the whole process of interfacial biocatalysis with CLSM to assess the stability of the conjugate cells at different times using CLSM. (c) Construction of reaction model for single-step catalysis based on the PIB platform. (d) Reaction profiles of Cal B from biphasic system (blue) and emulsion system (yellow); emulsion CLSM image to emphasize the stability of the emulsion system (inset). (e) Reaction profiles of BAL from biphasic system (blue) and emulsion system (yellow); emulsion CLSM image to emphasize the stability of the emulsion system (inset). (f) Reaction profiles of ATA from the biphasic system (blue) and emulsion system (yellow); emulsion CLSM image to emphasize the stability of the emulsion system (inset). (g) Reaction profiles of RR ADH from biphasic system (blue) and emulsion system (yellow); emulsion CLSM image to emphasize the stability of the emulsion system (inset).

Interfacial catalytic efficiency is well known to be significantly influenced by the emulsion's interfacial area.^7^ With this in mind, we overexpressed benzaldehyde lyase (BAL) in *E. coli* cells before forming the protein-cell conjugates, and utilized them for catalysis under various Pickering emulsion conditions. As shown in Fig. 3a, the catalytic efficiency initially increased and then decreased with increasing aqueous fraction, which aligns with the interfacial area results shown in Fig. S11.† We also labeled NaCas with FITC and grafted it onto cells overexpressing BAL cells to monitor the stability of the conjugates in real time during the catalytic process. The CLSM images revealed that both NaCas and the cells remained stable throughout the reaction, indicating the robustness of the system as well as the strong stabilization effect of the artificial display cells (Fig. 3b). On the other hand, the abundant and direct presence of whole cells at the emulsion interface offers a unique opportunity for relatively large-scale interfacial biocatalysis (as illustrated in Fig. 3c).

Additionally, we overexpressed *Candida antarctica lipase B* (Cal B) in *E. coli*, which was then modified with NaCas and used to form an emulsion for interfacial biocatalysis. For comparison, we used a biphasic system as a negative control, and operated under identical conditions, including the same number of *E. coli* cells. As shown in Fig. 3d, the catalytic efficiency of the protein-stabilized PIB system was substantially higher than that of the biphasic system. Specifically, the Cal B activity in the emulsion system was 33 times higher than in the biphasic system. Furthermore, we also performed an esterification reaction of fatty acids and fatty alcohols using *E. coli* overexpressing Cal B (Fig. S12†). The esterification reaction was easily activated and the cellular activity was still detectable even after 36 hours (Fig. S13†). These findings suggest that the increased interfacial area and the protective effect of the protein on the cell surface are the primary reasons for the significantly higher catalytic efficiency over reaction time.

Encouraged by these results, we investigated the versatility of *E. coli* as a platform for recombinant protein expression and extended the study to three additional enzymes: BAL, amine transaminase (ATA), and alcohol dehydrogenase (RR ADH). This allowed us to verify the generalizability of our strategy. For BAL, the emusion system successfully converted 100 mM of benzaldehyde to approximately 39.62 mM of benzoin, achieving an 80% yield, with high activity even after 12 hours (Fig. 3e). Similarly, for ATA and RR ADH, the catalytic efficiencies were above 80%, and the difference between the emulsion and biphasic systems was of an order of magnitude (Fig. 3f and g). Additionally, the artificial surface display-stabilized emulsion maintained interfacial morphology and cellular stability (Fig. S14†).

Therefore, we have demonstrated that the artificial surface display of protein-cell conjugates can serve as highly effective building blocks for the PIB platform, with strong potential for diverse biocatalytic applications.

### 2.4 Multienzyme cascade in interfacial biocatalysis

Cascade reactions are an elegant strategy in complex chemical synthesis, but their application is often limited by the challenges of complex purification and mutual deactivation of multiple catalysts.^38–40^ Given the unique properties of the protein-modified cell surfaces in our artificial display system, we aim to extend their use to cascade reactions in emulsion catalysis. To this end, we designed two different cascade reaction routes for interfacial catalysis.

**Fig. 4:**
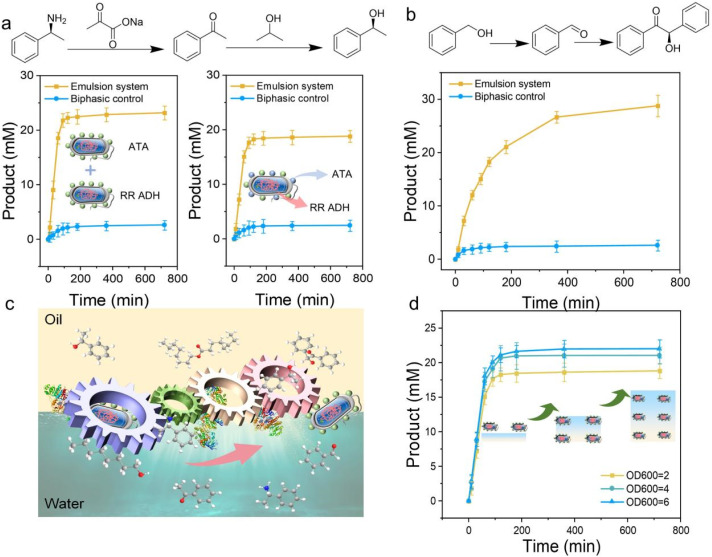
Investigation of multienzyme cascade reaction in protein-cell conjugates emulsion. (a) Cascade reaction catalyzed by ATA and RR ADH; emulsion systems stabilized with two conjugate cells overexpressing different enzymes, respectively (left); immobilization of ATA enzyme and NaCas on the surface of living cells directly for use as emulsifier particles (right). (b) Cascade reaction catalyzed by ADH ht and BAL. (c) Mass transfer processes at the water–oil interface. (d) Profiles of multienzymatic cascade reaction by conjugate cells with different OD_600_ values.

In the first cascade, we used two different approaches. First, we separately overexpressed amine transaminase (ATA) and alcohol dehydrogenase (RR ADH) in *E. coli* cells, and then engineered the cells with NaCas to form emulsions. Second, we coupled both ATA and NaCas directly onto the surface of *E. coli* cells, then formed emulsions. This design aimed to not only demonstrate the versatility of our system for cascade synthesis but also compare their effectiveness in catalysis. In both setups, 1-phenylethylamine was added to initiate the cascade reactions, where ATA converted it into acetophenone, which was then reduced by RR ADH to a chiral alcohol. As shown in Fig. 4a (left) and (right), both reactions achieved high cascade conversion, significantly outperforming the control experiments conducted in biphasic conditions. Interestingly, the cascade efficiency of the single protein-cell system, where one enzyme and NaCas were displayed on the same cell, was slightly lower than that of the multi-cell system with two intracellular enzymes in different cells. This may be due to differences in enzyme quantities between the systems, which are difficult to control considering the different preparation and multistep cascade. Nevertheless, we successfully demonstrated that cascade catalysis can be effectively enabled using different artificial surface display-based Pickering emulsions.

Building on the success of the first cascade reaction, we extended the approach to include more enzymes for cascade synthesis in emulsions. In the second cascade, we grafted *E. coli* cells overexpressing alcohol dehydrogenase (ADH ht) and BAL with NaCas and formed a stabilized emulsion system for benzoin production. As seen in Fig. 4b, the emulsion system displayed a significantly higher reaction rate compared to the biphasic system, with catalytic efficiency reaching 11 times that of the biphasic system. Importantly, we again monitored the stability of the living cells after the cascade reactions and found that the cells retained excellent stability, with no signs of inactivation or structural damage (Fig. S15†). We present this elegant process in Fig. 4c.

To further explore the tunability of the interfacial biocatalytic reaction, we conducted the same cascade reaction using emulsions formed with bacteria at different OD_600_ values. The results demonstrated that the reaction rate and yield were highly correlated with the OD_600_ value (Fig. 4d), indicating that protein-stabilized cells can be effectively scaled from single-step to multistep cascade reactions without compromising reactivity.^41,42^ Moreover, the reaction rate and yield can be nicely controlled by adjusting the OD_600_ value, making this strategy promising for large-scale applications.

### 2.5 Reusable protein-cell conjugates emulsion

**Fig. 5:**
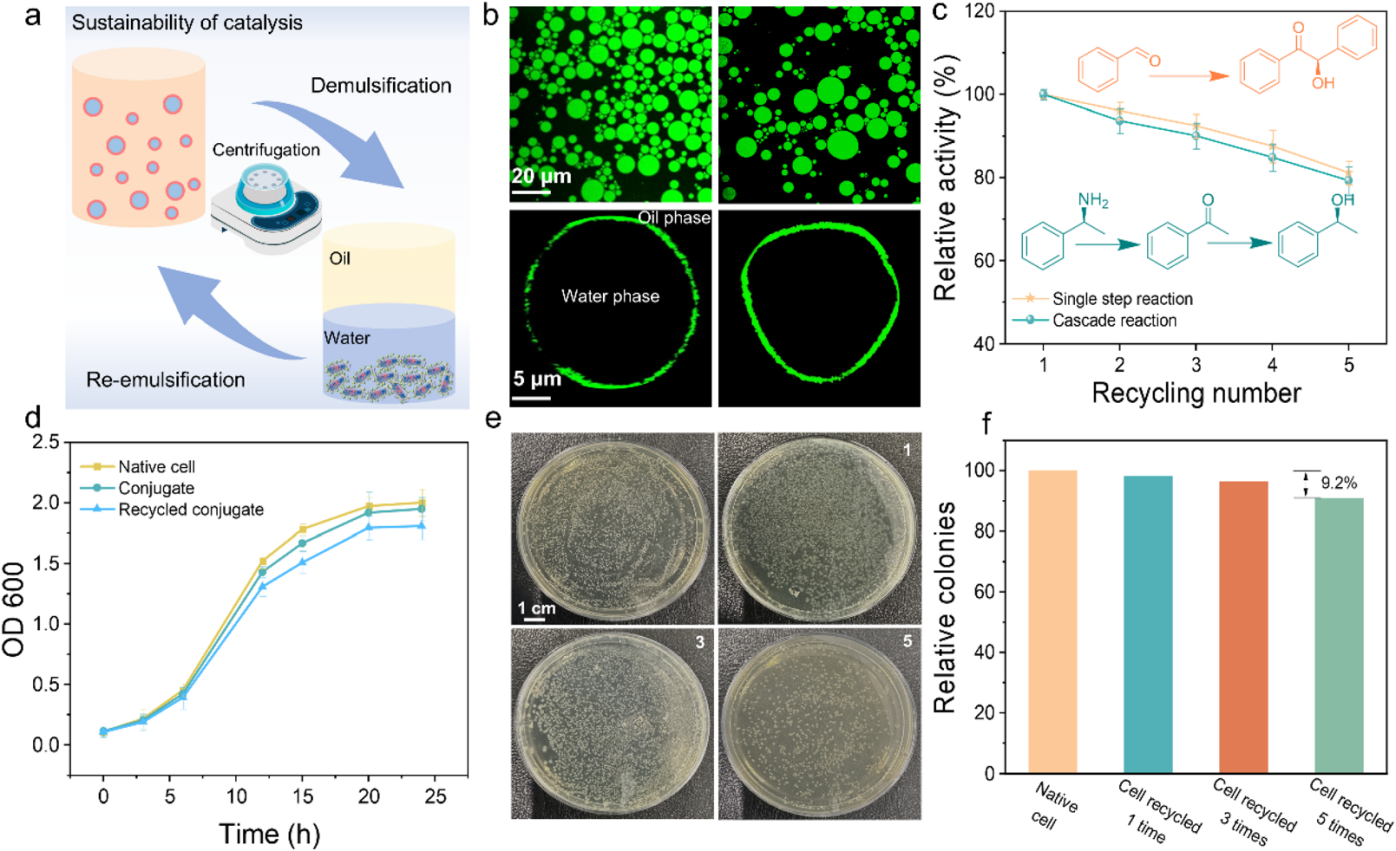
Sustainability of protein-cell conjugates emulsion. (a) Concept demonstration of sustainable interface catalysis. (b) CLSM images of the initial emulsion (left) and the emulsion after being recycled 5 times (right) to demonstrate recycling stability. (c) The single step reaction and the multi-enzyme cascade showed satisfactory high activity after recycling. (d) The cell growth curves of native cells (yellow), conjugates (green) and recycled conjugates (blue). (e) Plating assays of conjugate cells after the first recycled (Section 1), the third recycled (Section 3) and the fifth recycled (Section 5), comparing them with native cells (upper left section). (f) Relative colonies of conjugate cells after different times of recycled.

Today industry is increasingly focused on developing sustainable methods in green chemistry, with an emphasis on improving the atom economy.^43^ Given the unique biological structure and environmental resilience of protein-cell conjugates, we aimed to efficiently recycle and reuse living cells in interfacial catalysis. To this end, we performed recycling experiments where the emulsions were centrifuged and washed with water to recover the cells after each reaction. Remarkably, we were able to reform stable emulsions using the recovered cells (Fig. 5a).

We then evaluated the stability of the emulsions before and after recycling under a microscope. As shown in Fig. 5b, the emulsion's microscopic morphology remained largely unchanged even after five recycling cycles, with cell conjugates still uniformly distributed at the oil–water interface. Additionally, the data indicated that after five cycles, enzymes in cells retained over 80% of their activity (Fig. 5c and S16†), and the yield of the reaction decreased by only 13.5% (Fig. 5b). This slight decline was likely due to the partial loss of cells during centrifugation and repeated operations.

To further demonstrate the robust proliferative capacity of the recycled protein-cell conjugates, we compared the growth curves of native cells, conjugate cells, and conjugate cells recycled five times. Surprisingly, there were no significant differences in their growth rates (Fig. 5d). Moreover, when we cultured the recycled cells on plate, even after five recycling cycles, they still exhibited excellent proliferative activity, with colony numbers comparable to those of native cells (Fig. 5e and f). These findings provide strong evidence that the NaCas-stabilized PIB system does not impair the intrinsic proliferative ability of living cells, making them highly reusable for future catalytic applications.

## 3 Conclusion

In summary, we present an artificial surface display strategy that leverages the simple coupling of sodium caseinate (NaCas) to living cells, accordingly creating protein-cell conjugates for robust Pickering interfacial biocatalysis (PIB). The obtained conjugates provide dual functionality: protecting cells from various environmental stresses while enhancing their amphiphilicity, which allows the formation of stable emulsions. The high catalytic efficiency at the oil–water interface facilitates the scalability of the platform from single-step reactions to multistep cascade reactions with minimal loss in enzyme activity.

Furthermore, the approach offers several other advantages, including simplified preparation without the need for protective coatings, broad applicability in different biocatalytic scenarios, and the ability to accommodate multiple enzymes on the cell surface, enabling efficient cascade catalysis. Notably, our PIB platform demonstrates strong recyclability, with conjugated cells retaining over 80% of catalytic activity even after five reuse cycles. This high reusability, combined with stable performance under harsh conditions, underscores the system's potential for sustainable and cost-effective biocatalytic processes.

## Data availability

All data are available in the main text and the ESI† or available from the authors upon reasonable request.

## Author contributions

C. W. and Z. W. conceptualized and supervised the project. X. W. performed the experiments. H. K. provided guidance and supervision related to the microbiological work. C. W., X. W. and Z. W. wrote the paper.

## Conflicts of interest

We declare that we do not have any commercial or associative interest that represents a conviction of interest in connection with the work submitted.

## Supplementary Material

SC-OLF-D4SC08063G-s001
