# Supplementary material for: Protein-cell conjugates as artificial surface display for interfacial biocatalysis
Source: Chem Sci. 2025 Feb 11;16(11):4892–9. doi: 10.1039/d4sc08063g (PMC11811893; doi:10.1039/d4sc08063g)
Supplement: SC-016-D4SC08063G-s001 [file SC-016-D4SC08063G-s001.pdf]

Supplementary Information (SI) for Chemical Science.
This journal is © The Royal Society of Chemistry 2025

Protein-cell conjugates as Artificial Surface Display for Interfacial Biocatalysis

Xiankun Wu [1,2], Henrik Karring [3], Zhongkai Wang [1*], Changzhu Wu [2,4*]

[1] Anhui Provincial Engineering Center for High Performance Biobased Nylons, School of Materials and Chemistry, Anhui Agricultural University, Hefei, Anhui 230036, China

[2] Department of Physics, Chemistry and Pharmacy, University of Southern Denmark, Campusvej 55, 5230 Odense, Denmark

[3] Department of Green Technology, University of Southern Denmark, Campusvej 55, 5230 Odense, Denmark

[4] Danish Institute for Advanced Study (DIAS), University of Southern Denmark, Campusvej 55, 5230 Odense, Denmark

* Correspondence:

Email: wu@sdu.dk (C.W.), wangzk6@ahau.edu.cn (Z.W).

Table of Contents

Experimental procedures.. 3

Materials and instruments... 3

Preparation of *NaCas - E. coli* conjugates.. 3

Expression of Candida antarctica Lipase B (Cal B)... 4

Expression of benzaldehyde lyase (BAL)... 4

Expression of green fluorescence protein (GFP).. 4

Expression of alcohol dehydrogenase from Rhodococcus ruber (RR ADH)……….. 5

Expression of alcohol dehydrogenase from Bacillus stearothermophilus (ADH ht).. 5

Expression of amine transaminase (ATA)... 5

Preparation and purification of ATA enzyme.. 5

Viability assay of E. coli cells... 6

Plating assay of E. coli cells... 6

Emulsions with different water-to-oil ratios... 6

Single step interfacial catalysis with different *E. coli*.. 6

Multienzyme cascade catalysis... 6

Re-culturing and growth curve of E. coli cells.. 7

Recycling experiments.. 7

Supplementary results.. 8

Reference... 18

Experimental procedures

Materials and instruments

All the chemicals and reagents were used without further purification unless mentioned. 1-(3-Dimethylaminopropyl)-3-ethylcarbodiimide (EDC), N-Hydroxysuccinimide (NHS), Eosin Y, Fluorescein isothiocyanate isomer I (FITC) were purchased from Sigma Aldrich. Sodium chloride, Sodium bicarbonate, and Sodium Caseinate (NaCas) was purchased from VWR. Propidium iodide and SYTO 9 were purchased from Thermo Fisher Scientific. All other reagents were of analytical grade.

Gas Chromatography (GC) analysis was carried out on a Shimadzu Nexis™ GC-2030 gas chromatography machine, equipped with an AOC-20i/s autoinjector/autosampler.

UV-visible spectra were performed using a Cary series UV-Vis-NIR spectrophotometer (Agilent Technologies) at room temperature using a 2 mm quartz glass.

Epifluorescent images of cells were acquired using a Nikon A1 confocal microscope with NIS-Elements AR 5.30 software. Channels were set up in the 407 nm (DAPI) and 561 nm (TRITC) range using a CoolLED "pE-300 white" light source, standard DAPI and TRITC filter cubes and an Andor Zyla monochrome camera. The contrast was adjusted to the desired levels in ImageJ ver. 1.53v.

Matrix-assisted laser desorption/ionization time-of-flight (MALDI-TOF) mass spectra were acquired using a MALDI-TOF UltrafleXtreme-II tandem mass spectrometer (Bruker) operating in positive ion linear mode. The samples were prepared on an MTP 384 polished steel target plate with a matrix Super-DHB. The matrix was a 9:1 mixture of 2, 5-dihydroxybenzoic acid (DHB) and 2-hydroxy-5-methoxybenzoic acid, prepared at a 50 mg mL^{-1} in a 1:1 solution of Millipore water and acetonitrile, with 0.1% trifluoroacetic acid.

Preparation of NaCas - E. coli conjugates

EDC (16 mM) and NHS (10 mM) were added to 10 mL of 40 mg/mL NaCas solution. The freshly collected *E. coli* was dispersed in 40 mL KPI buffer (pH 8.0, 0.1 M) to reach a final OD_{600} of 2.0. The crude product was obtained by adding 10 mL of the *E. coli* dispersion to the NaCas solution and reacting for 6 h at 4 °C. After the reaction,

the extra free NaCas and unreacted substrate were removed by washing 3 times with KPI buffer (pH 8.0, 0.1 M). The final conjugates were suspended in a KPI buffer (pH 8.0, 0.1 M) to reach a final OD_{600} of 2.0 for further use.

Expression of Candida antarctica Lipase B (Cal B)

Expression of Cal B was according to the following protocol: preculture of *E. coli* strain BL21 (DE3) containing pET22b_Cal B-His (after expression Cal B is localized in the periplasm) was employed as Strain. Preculture was incubated in lysogeny broth (LB) medium with 100 μg/mL ampicillin at 37 °C overnight. Mainculture was incubated in the autoinduction medium with 100 μg/mL ampicillin at 20 °C overnight[1].

Auto-Induction Medium (2 L): 20 g Tryptone, 10 g Yeast extract, 40 mL 50 xM stock solution, 4 mL 1M $MgSO_4$, 0.4 mL 1000x Trace Metals stock solution, 40 mL 50x 5052 stock solution.

Add sterilized stocks to autoclaved Tryptone + Yeast medium right before use.

50x salt stock solution: 1.25 M Na_2HPO_4X7 H_2O, 1.25 M KH_2PO_4, 2.5 M NH_4Cl, 0.25 M Na_2SO_4.

50X5052 stock solution: 25% Glycerol, 2.5% Glucose, 10% G-Lactose.

1000x stock solution of trace metals: 50 mM $FeCl_3$, 20 mM $CaCl_2$ (x2 H_2O), 10 mM $MnCl_2$ (x4 H_2O).
10 mM $ZnSO_4$ (x7 H_2O), 2 mM $CoCl_2$ (x6 H_2O), 2 mM $CuCl_2$, 2 mM $NiCl_2$, 2 mM Na_2MoO_4, 2 mM Ma_2SeO_3, 2 mM H_3BO_3.

Expression of benzaldehyde lyase (BAL)

The BAL was produced with the recombinant strain *E. coli* BL21 (DE3) containing pET28a plasmid[2]. The expression procedure is similar to the previous literature. As the preculture, *E. coli* cells were incubated in an LB/kanamycin medium overnight at 37 °C. The preculture was then transferred to the main culture and incubated at 37 °C until reaching OD_{600} = 0.6, whereby a final concentration of 0.8 mM isopropyl β-D-1-thiogalactopyranoside (IPTG) was added to induce the expression. After that, further incubation was carried out for another 16 h. The cells were collected by centrifugation (4 °C, 5000 rpm, 10 min).

Expression of green fluorescence protein (GFP)

GPF in *E. coli* cells was expressed using the strain *E. coli* BL21 (DE3) containing the plasmid pET28b-GFP-His. According to previous research, the preculture was prepared with Luria-Bertani medium (LB) containing 50 μg mL^{-1} kanamycin and recombinant strain *E. coli* was grown at 37 °C while shaking at 220 rpm overnight, and then the preculture was transferred into the main culture with 50 μg mL^{-1} kanamycin. Isopropyl β-D-1-thiogalactopyranoside (IPTG) (0.5 mM) was added to induce the GFP expression at 25 °C overnight when OD_{600} reached 0.8-1.0 for the main culture. The bacteria pellets were obtained by centrifugation (4 °C, 5000 rpm, 10 min).

Expression of alcohol dehydrogenase from Rhodococcus ruber (RR ADH)

First, a preculture was prepared by incubating *E. coli* BL21 containing pET22b+-RR ADH plasmid in LB medium with 100 μg/mL ampicillin overnight at 37 °C. Second, a main culture was prepared in LB medium with 100 μg/mL ampicillin at 37 °C until reaching OD_{600} = 0.6, followed by adding 1 mM IPTG and 0.43 mM $ZnSO_4$ to induce the RR ADH expression. After further being incubated overnight at 25 °C, cells were collected by centrifugation (4 °C, 5000 rpm, 10 min).

Expression of alcohol dehydrogenase from Bacillus stearothermophilus (ADH ht)

ADH-ht in E. coli cells was expressed according to a previous publication[3]. In a typical procedure, *E. coli* strain BL21 containing plasmid pET21a+-ADH-ht plasmid was incubated overnight at 37 °C as the preculture. The preculture was then transferred to the main culture and incubated at 37 °C until reaching OD_{600} 0.7, whereby a final concentration of 1 mM isopropyl β-D-1thiogalactopyranoside (IPTG) was added to induce the expression. The cultures were shaken overnight at 20 °C, 120 rpm. After that, the bacteria pellets were obtained by centrifugation (4 °C, 5000 rpm, 10 min).

Expression of amine transaminase (ATA)

A preculture was prepared by incubating *E. coli* BL21 containing pET22b-ATA plasmid in terrific broth (TB) medium with 100 μg/mL ampicillin overnight at 37 °C. Then a main culture was prepared in a TB medium with 100 μg/mL ampicillin at 37 °C until reaching OD_{600} = 0.6, followed by adding 1 mM IPTG to induce the ATA

expression. After further being incubated overnight at 25 °C, cells were collected by centrifugation (4 °C, 5000 rpm, 10 min).

Preparation and purification of ATA enzyme

To get free ATA enzyme, the cell pellets were resuspended in lysis buffer using HEPES buffer (Mw 238.3 g/mol; pH 7,5 and 50 mM) and adding 0.1 mM pyridoxal 5'-phosphate (PLP), and then the DNAse I was added into suspension with 1 h incubation at room temperature for DNA degradation. After ultrasonication (3 min, 30 % amplitude, 5 s pulse, 20 s pause) while keeping the cells on ice the resulting lysate was centrifuged (4 °C, 12000 rpm, 60 min) to get clear supernatant including crude ATA after ultrasonication (3 min, 30 % amplitude, 5 s pulse, 20 s pause) under ice bath. The crude ATA was further purified on an ÄKTA pure chromatography system with a HiPrep™ 16/10 column to get pure ATA followed by lyophilization to store at -20 °C for further use.

Viability assay of E. coli cells

Cells (native cells, conjugate cells and conjugate cells after 5 times recycling) were respectively dispersed in a KPI buffer (pH 8, 0.1 M). Then cells were incubated with SYTO 9 for 20 min, followed by incubation with propidium iodide for another 20 min and washed three times with KPI buffer (pH 8, 0.1 M). After that, fluorescent microscopy was used to measure cell viability.

Plating assay of E. coli cells

Cells (native cells, conjugate cells and conjugate cells after 5 times recycling) were respectively suspended in 1 mL KPI buffer (pH 8, 0.1 M) to reach a final OD_{600} of 0.3. 30 μL of cell suspension was taken and put on an LB agar plate. The plates were further incubated at 30 °C for 48 h.

Emulsions with different water-to-oil ratios

To optimize interfacial biocatalysis, we prepared emulsions with different water-to-oil ratios. Typically, 1 mL conjugate cells were first taken from the stock solution, collected by centrifugation, and then used as emulsifiers. By adding different amounts of water and organic solvents to a total volume of 2 mL, emulsions were obtained with gentle shaking by hand. These different emulsions were then applied to catalysis reactions. The reactions were initiated upon providing a final concentration of 100 mM substrates. At each time interval, 20 μL emulsion was withdrawn and extracted with 190 μL ethyl acetate. The ethyl acetate phase was dried with anhydrous

magnesium sulfate. After centrifugation, 150 µL supernatant was subjected to gas chromatography (GC) analysis. The experiment was repeated in triplicate.

Single step interfacial catalysis with different E. coli

First, 1 ml of *NaCas* - *E. coli* KPI solution overexpressing different enzymes was mixed with 1 mL of organic solution and the emulsion was made by handshaking. Then, 100 mM substrate was added in emulsions to initiate the reaction. At each time interval, a 20 µL mixture was taken and extracted with 190 µL ethyl acetate, followed by drying with anhydrous magnesium sulfate. After centrifugation, 150 µL supernatant was withdrawn for GC measurements. The experiment was carried out in triplicate. To obtain the highest catalytic efficiency, all single-step experiments were carried out for 12 h except for the esterification reaction. Given the activity of the enzyme-catalyzed esterification reaction, we have extended the time for the Cal B-activated esterification reaction to 36 h.

Multienzyme cascade catalysis

To compare the differences, we performed a multi-enzyme cascade catalysis reaction with two methods.

i) First, we used living cells overexpressing ATA and RR ADH with 1 ml of organic solvent to form an emulsion by hand-shaking. Then 100 mM of substrate was added to initiate the reaction. At each time interval, a 20 µL mixture was taken and extracted with 190 µL ethyl acetate, followed by drying with anhydrous magnesium sulfate. After centrifugation, 150 µL supernatant was withdrawn for GC measurements. The experiment was carried out in triplicate.

ii) First we immobilized free ATA enzyme on the surface of living cells overexpressing RR ADH. Then 1 mL of organic solvent was added to form an emulsion by hand shaking directly. The reaction was initiated after the addition of 100 mM of α-methylbenzylamine. At each time interval, a 20 µL mixture was taken and extracted with 190 µL ethyl acetate, followed by drying with anhydrous magnesium sulfate. After centrifugation, 150 µL supernatant was withdrawn for GC measurements. The experiment was carried out in triplicate.

Re-culturing and growth curve of E. coli cells

Cells after recycled were suspended in 1 mL LB medium containing 100 µg/mL kanamycin to reach a final OD_{600} of 0.1 and incubated at 37 °C. Afterwards, UV-

visible spectroscopy was then used to measure OD_{600} at different time intervals. The experiments were performed in triplicate.

Recycling experiments

The recycling procedure was the same for both single step catalysis and multi-enzyme cascades. After the first catalysis, the emulsion system was demulsified in a centrifuge (6000 rpm, 5 min) to obtain the protein-cell conjugates, which were washed three times with KPI buffer (pH=8). The clean conjugates were dispersed with the same volume of KPI (pH=8) buffer until the OD_{600} was approximately 0.2. Subsequently, toluene was poured proportionally and the emulsion was formed again by hand shaking for the next catalytic reaction. The recycling procedures are similar each time.

Supplementary results

Table S1 The effect of different coupling agent concentrations on the retaining activity of the couplers when applied to E. coli cells.

Items	Product(mM)	Retaining activity (%)
Native cell	28.64	100
Conjugate-EDC (8mM) +NHS (6mM)	27.79	97.04
Conjugate-EDC (16mM) +NHS (10mM)	27.42	95.75
Conjugate-EDC (25mM) +NHS (15mM)	25.47	88.93
Conjugate-EDC (35mM) +NHS (20mM)	22.11	77.19

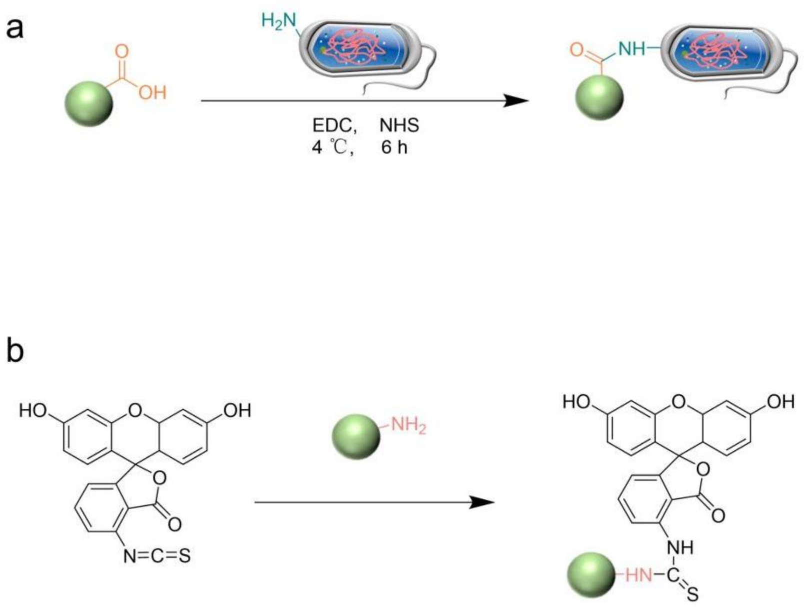

Fig. S1 a) Synthesis route of living cell conjugates, b) staining process of FITC with NaCas (green sphere).

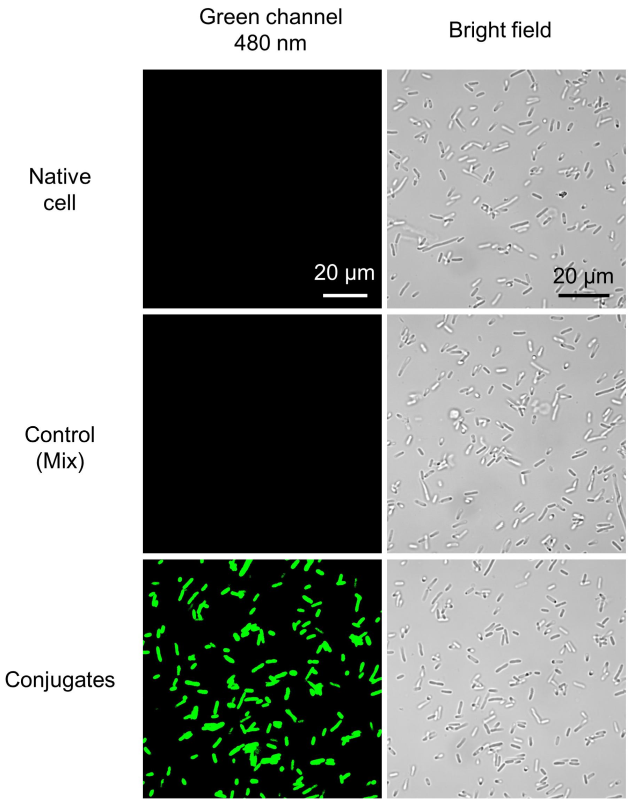

Fig. S2 Optical and fluorescence images of the native cell, the mixture of NaCas-FITC and E. coli (Mix) and NaCas-conjugated cells (Conjugates).

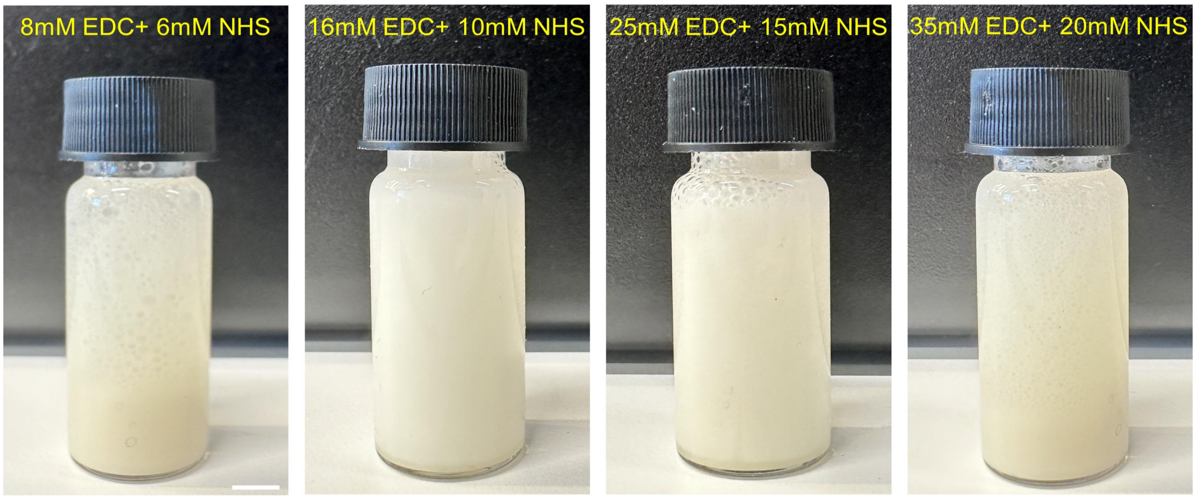

Fig. S3 Macrophotographs of emulsions formed by conjugates prepared with different amounts of coupling agents.

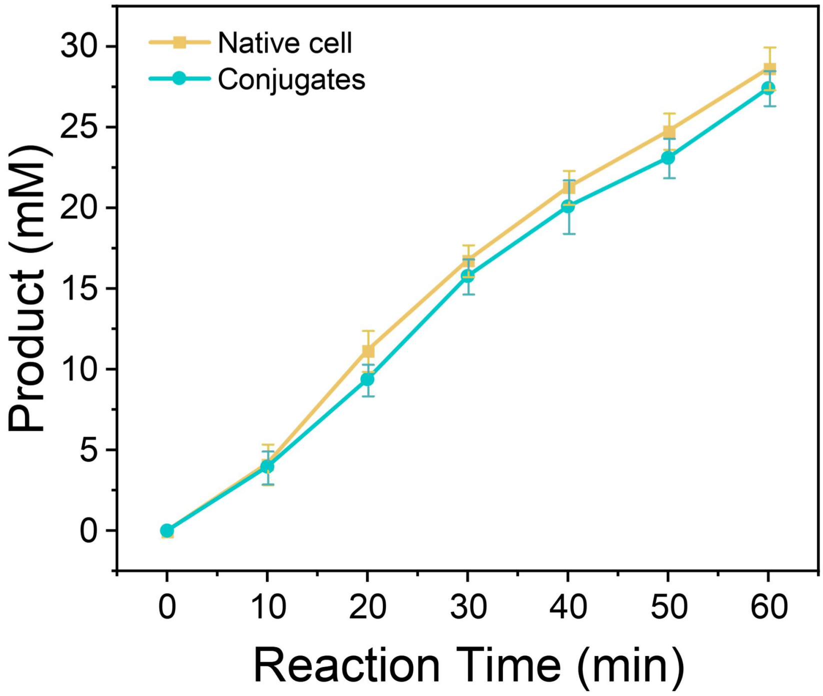

Fig. S4 Native cells and conjugate cells exhibit similar catalytic activity.

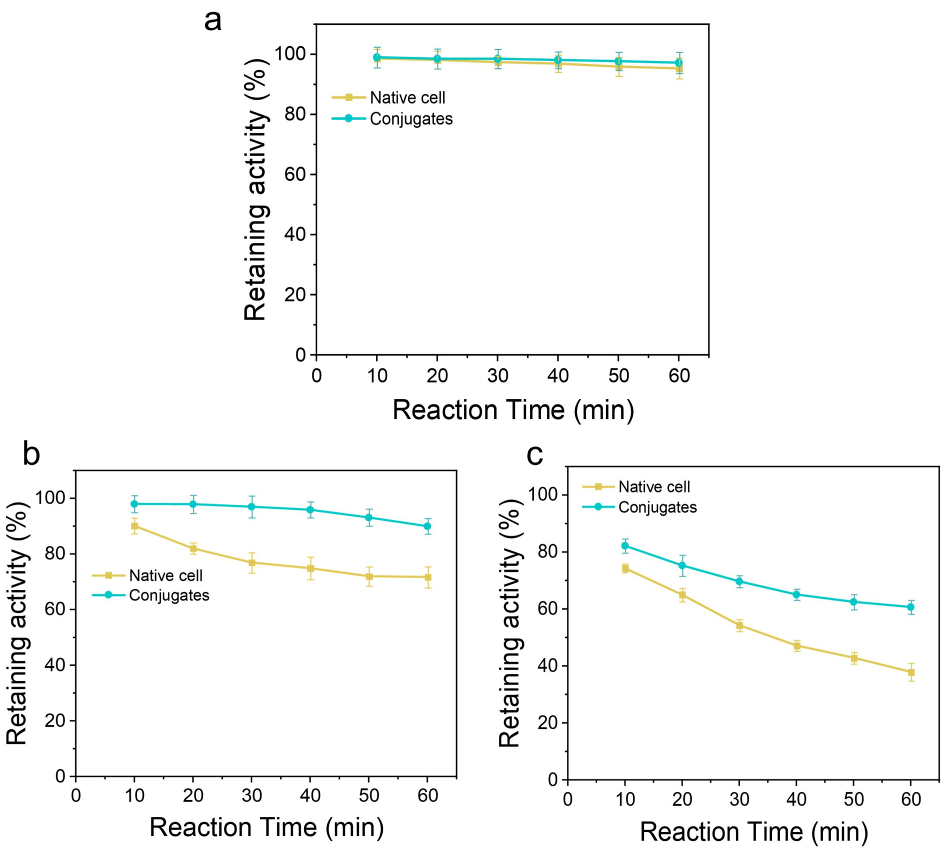

Fig. S5 Retaining activity of native cells and protein-cell conjugate exposure to different temperatures for 1 h.

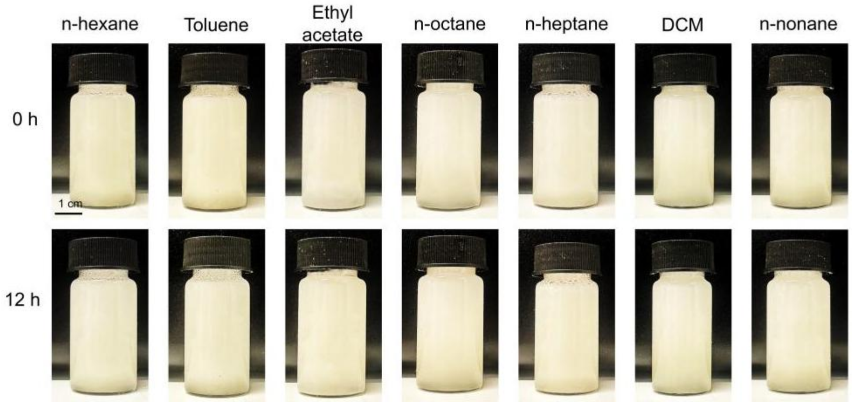

Fig. S6 Emulsion systems formed by protein-cell conjugates with different organic solvents (upper), the emulsion can be stabilized for at least 12 hours (lower).

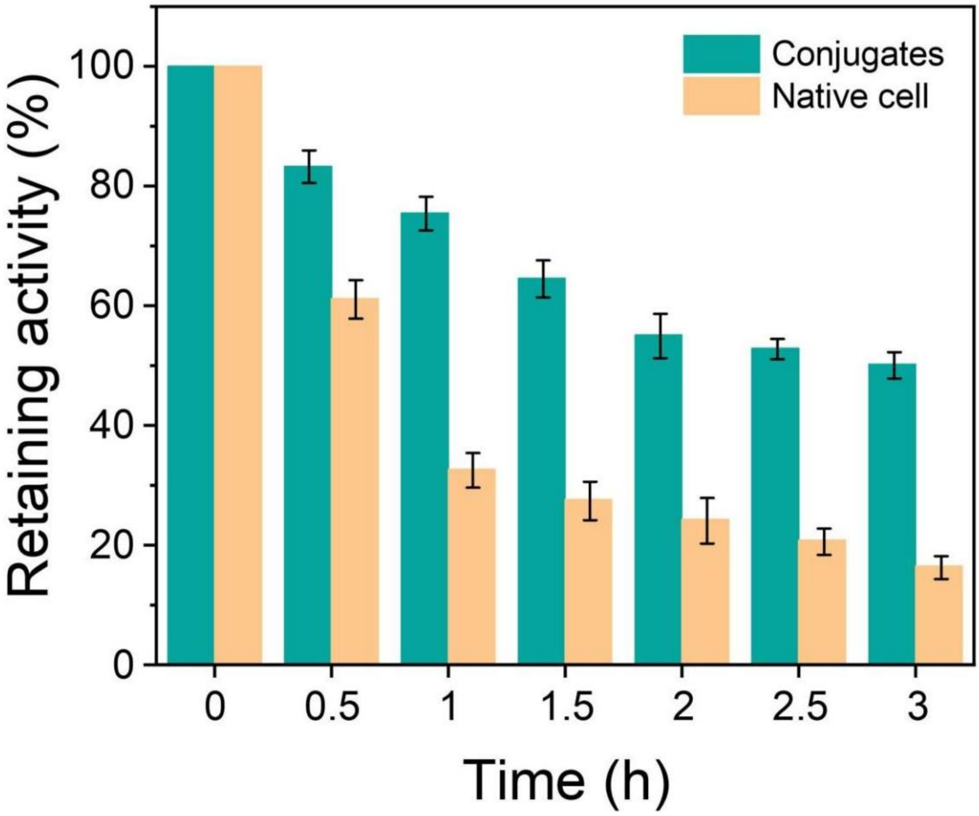

Fig S7 Protein-cell conjugates exhibit excellent resistance in toluene.

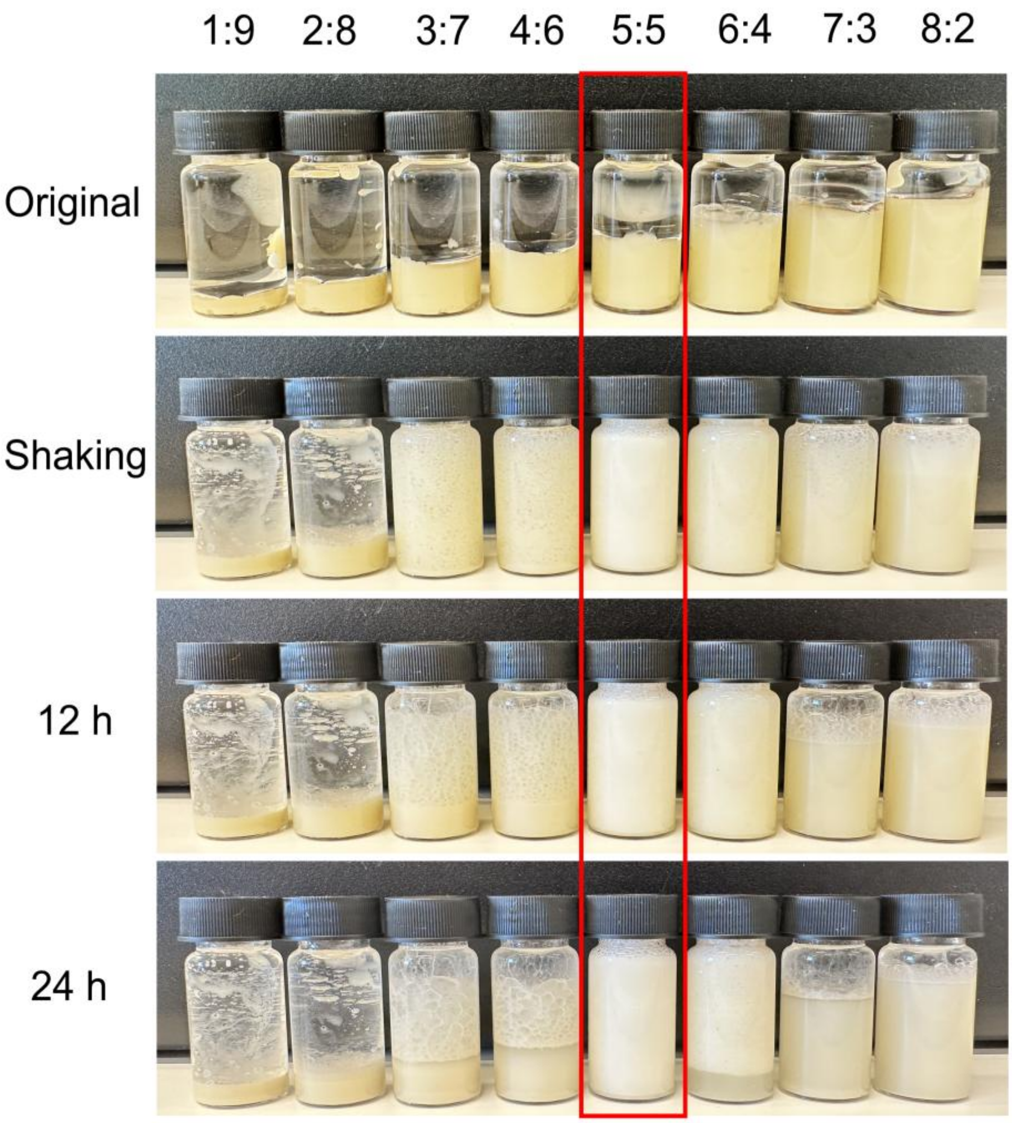

Fig. S8 Images of emulsion systems with different water-to-oil ratios after handshaking and standing for different periods.

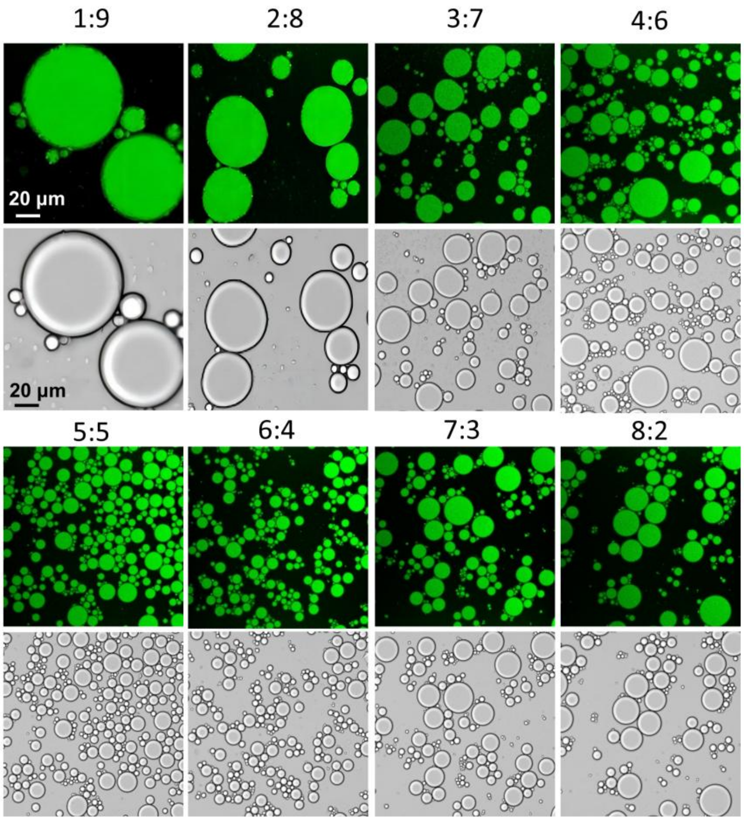

Fig. S9 The fluorescence microscopy images of emulsion droplets formed by conjugates with different water-to-oil ratios from 1:9 to 8:2.

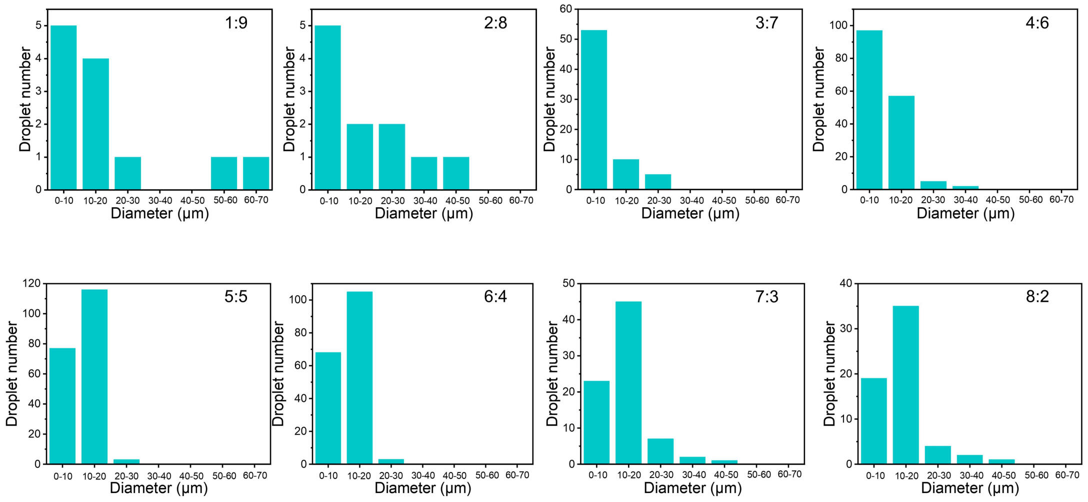

Fig. S10 The number and size distribution of emulsion droplets formed by conjugates with different water-to-oil ratios from 1:9 to 8:2.

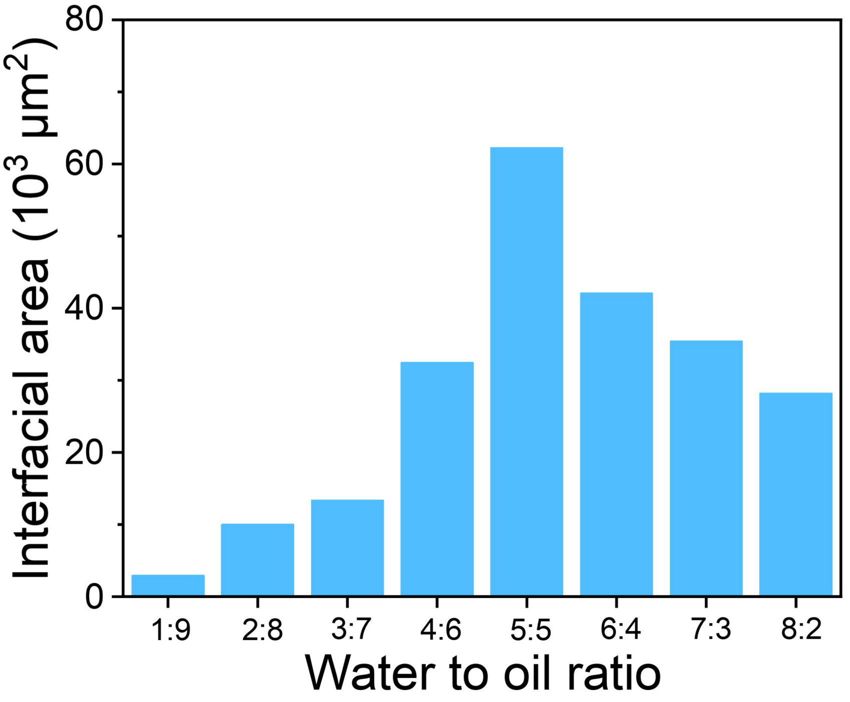

Fig. S11 Interfacial area calculation for all emulsion components.

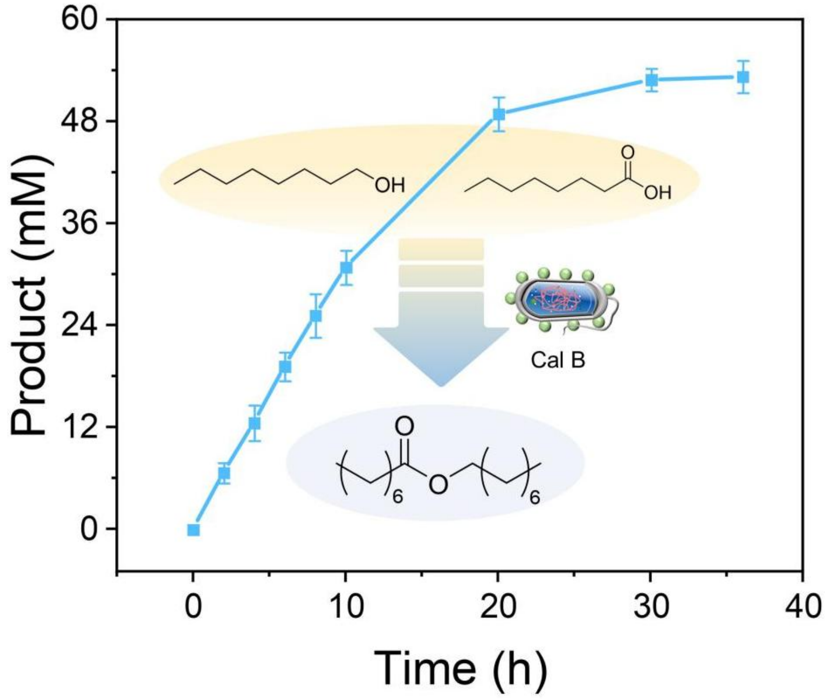

Fig. S12 Cal B-catalyzed esterification reactions to emphasize the compatibility of the PIB platform with multiple substrates.

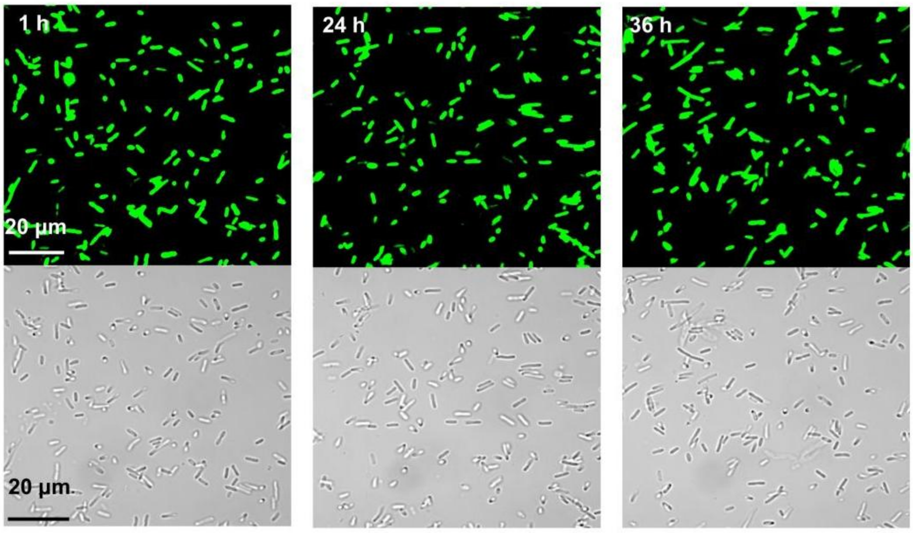

Fig. 13 Examination of the whole process of multienzymatic cascade reaction with CLSM to assess the stability of the conjugate cells.

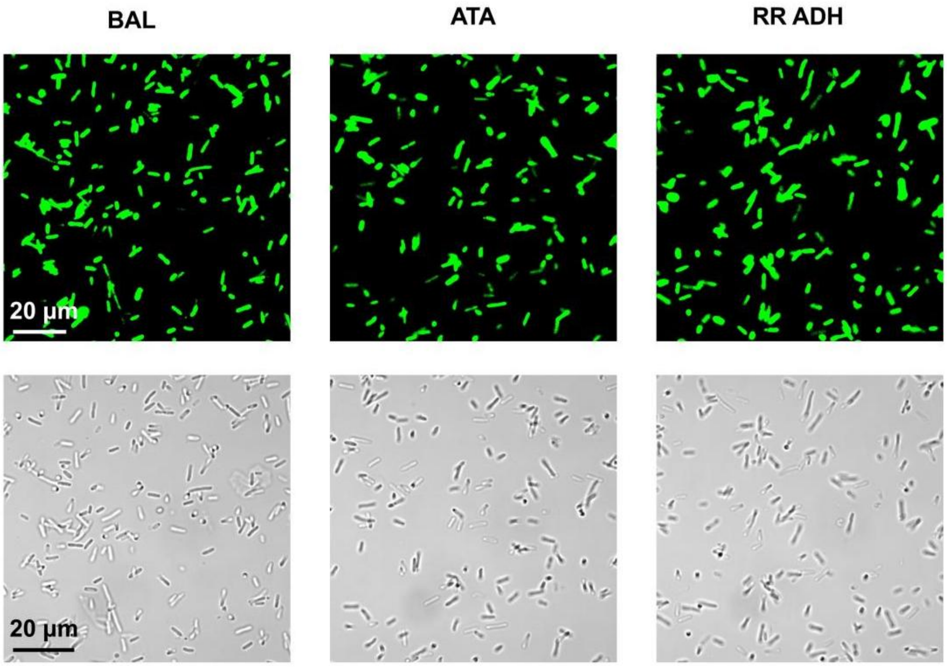

Fig. S14 Fluorescence images of living *E. coli* cells overexpressing different enzymes at the end of a single-step catalysis reaction.

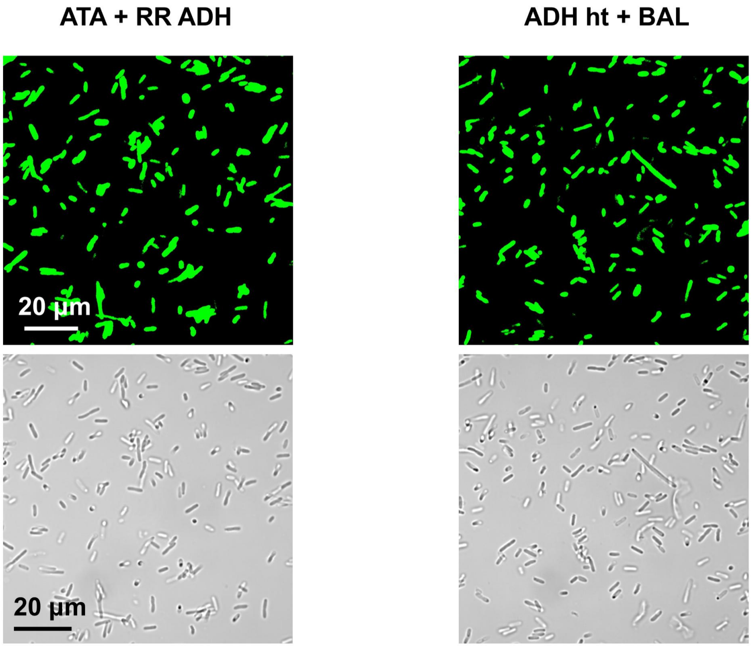

Fig. S15 Fluorescence images of living *E. coli* cells after two different cascade catalysis reactions.

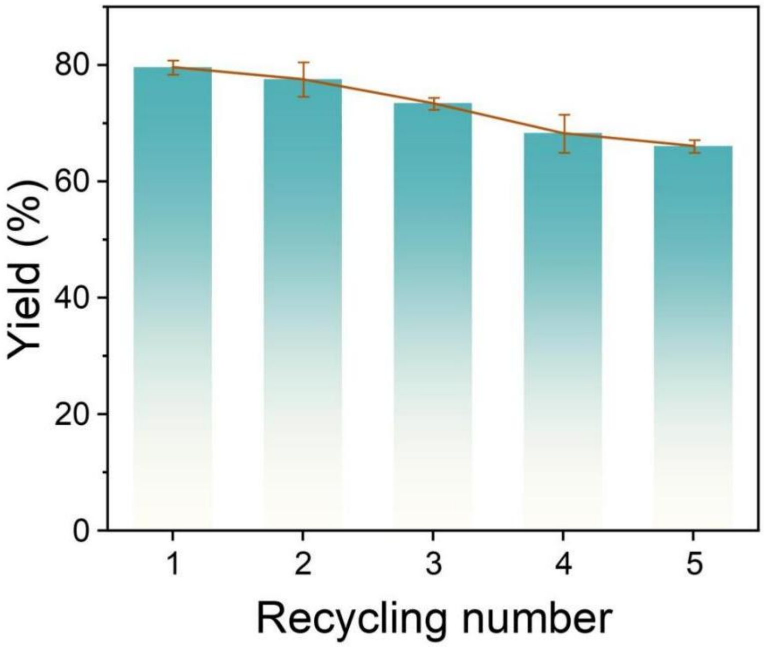

Fig. S16 Yield of interface catalysis reactions of protein-cell conjugates after 5 recycling times showed an insignificant difference from the initial sample.

Reference

1 Z. Sun, R. Hubner, J. Li, C. Wu, *Nat Commun* 2022, **13**, 3142.

2 Z. Sun, U. Glebe, H. Charan, A. Boker, C. Wu, *Angew Chem Int Ed Engl* 2018, **57**, 13810-13814.

3 J. H. Sattler, M. Fuchs, F. G. Mutti, B. Grischek, P. Engel, J. Pfeffer, J. M. Woodley, W. Kroutil, *Angew Chem Int Ed Engl* 2014, **53**, 14153-14157.
